# A Fully-Flexible Solution-Processed Autonomous Glucose Indicator

**DOI:** 10.1038/s41598-019-43425-x

**Published:** 2019-05-06

**Authors:** Jonathan D. Yuen, Ankit Baingane, Qumrul Hasan, Lisa C. Shriver-Lake, Scott A. Walper, Daniel Zabetakis, Joyce C. Breger, David A. Stenger, Gymama Slaughter

**Affiliations:** 1 Center for Bio-Molecular Science and Engineering, U.S. Naval Research Laboratory, Washington, DC 20375 USA; 2 Frank Reidy Research Center for Bioelectrics and Department of Electrical & Computer Engineering, Old Dominion University, Norfolk, VA 23529 USA; 3 Department of Computer Science and Electrical Engineering, University of Maryland Baltimore County, Baltimore, MD 21250 USA

**Keywords:** Devices for energy harvesting, Electronic devices, Sensors and biosensors

## Abstract

We present the first demonstration of a fully-flexible, self-powered glucose indicator system that synergizes two flexible electronic technologies: a flexible self-powering unit in the form of a biofuel cell, with a flexible electronic device - a circuit-board decal fabricated with biocompatible microbial nanocellulose. Our proof-of-concept device, comprising an enzymatic glucose fuel cell, glucose sensor and a LED indicator, does not require additional electronic equipment for detection or verification; and the entire structure collapses into a microns-thin, self-adhering, single-centimeter-square decal, weighing less than 40 mg. The flexible glucose indicator system continuously operates a light emitting diode (LED) through a capacitive charge/discharge cycle, which is directly correlated to the glucose concentration. Our indicator was shown to operate at high sensitivity within a linear glucose concentration range of 1 mM–45 mM glucose continuously, achieving a 1.8 VDC output from a flexible indicator system that deliver sufficient power to drive an LED circuit. Importantly, the results presented provide a basis upon which further development of indicator systems with biocompatible diffusing polymers to act as buffering diffusion barriers, thereby allowing them to be potentially useful for low-cost, direct-line-of-sight applications in medicine, husbandry, agriculture, and the food and beverage industries.

## Introduction

The increasing connectivity of physical objects to the virtual network, as mediated by electronics representing the Internet-of-Things (IoT), is merely at its incipience^1–3^. It is estimated that by 2020, there will be 30 billion IoT-related devices, wherein the global IoT market is projected to reach $7.1 trillion^4^. Flexible electronics are poised to play an essential role in this expansive social and technological development due to their integrability^5–7^. Unlike typical “hard” electronics which are objects-in-themselves, flexible electronics, particularly imperceptible electronics, can be envisioned as minimalized objects that can be seamlessly integrated or affixed onto other physical objects unobtrusively and without hindrance.

However, a few technical issues impede the effective integration of flexible with the IoT, of which energy autonomy is of particular interest^8–10^. We define autonomous energy/power sources as ones which draw energy from the devices themselves or from the objects which they are attached to, i.e., not from external sources of energy such as power outlets or the sun. As more and more sensors are located in remote, hard to access places, or placed on autonomous/mobile systems, such as the human body, sensor nodes that can operate without external energy supply become vital^11–13^. Unfortunately, most of the currently used power systems for flexible electronics involve rigid batteries, which presents a series of obvious problems, such as frequent charging and battery replacement, and wearable capability.

In order to make flexible electronics truly operationally autonomous, we need to combine a flexible electronic device with a flexible power source in order to create an integrated energy-autonomous flexible electronic system. Fully developed examples of flexible electronics or flexible power sources have been presented^14,15^, but until now, deficiencies in flexible power technology have prevented the successful integration of the two technologies. Flexible batteries and supercapacitors require frequent external charging and/or replacement, and hence are not autonomous^16,17^. Radiation-powered sources, such as solar cell or wireless antennas require a constant external energy source which the sun or RF cannot provide^18,19^. Flexible thermoelectric sources require high temperature differentials to produce sufficient power^20,21^, whereas triboelectric or piezoelectric power sources require the object it is attached to be in constant and high motion^22–24^.

Although a few examples of fully-flexible, self-powered devices exist, their applications have utility only within a narrow spectrum. These devices involve the *in-situ* conversion of one form of energy (i.e., mechanical) into electrical energy in order to power small electronics devices. One incarnation involves coupling flexible triboelectric or piezoelectric power sources with LEDs on flexible substrates^25–27^; or more intriguingly, arrays of nanostructured integrated semiconducting heterostructures that contain both piezoelectric and light emission components on flexible substrates^28^. Applications for touchscreens or motion sensors have been suggested, but utility is limited as intimate contact is required and additional electronics are required to interpret the optical output in order to interface with other systems^29,30^. Another interesting demonstration is a flexible PZT piezoelectric device that is used directly as a pacemaker^31^. However, an external mechanical force is required to stimulate electrical pulses, and the high cost of PZT and the cost of transferring an ultra-thin sheet of PZT onto a flexible substrate will prevent wide-scale utility of this technology. In contrast, the technology described here uses standard electronic components and is completely manufactured under ambient conditions, making it a low-cost technology.

In this report, we present one of the first examples of a fully autonomous, self-powering flexible electronic device that can be used to indicate the presence of an analyte. The self-powering component is based on a biofuel cell which provides electrical power via the enzyme-catalyzed oxidation of glucose; this in turn powers a flexible PCB indicator, in this case a light-emitting diode (LED). The LED is powered by the charging/discharging of electrical energy in a capacitor using a charge pump circuit. In addition to energy autonomy, another important goal that was achieved with this device is operational autonomy, that is, the device does not need to be connected to other electronic equipment to interpret its data output. Instead, processed output can be directly sensed by the user (through visual, tactile, audio, etc. means), or wirelessly with electronic devices, thereby freeing the “wearer” of the flexible device from the burden of additional apparatus. Work done in this area has so far been limited to forming a flexible biofuel cell, with no focus in the development of flexible circuitry or electronics to couple with the cell. Such cells are either connected to a potentiostat^32^ or a custom-manufacture printed circuit board^33,34^ and therefore the present device is the first example of a fully-flexible self-powered glucose indicator. The present proof-of-concept fabrication may have applications in providing immediate, round-the-clock glucose monitoring for diabetes which in 2013 resulted in 75,578 deaths in the United States. While the present fabrication focuses on glucose indication, the enzyme cascade that dictates the biofuel cell operation can be modified to be attuned to other analytes such as lactate in mammals, or sugars in plants and comestibles, with potential applications in diverse industries as in medical and husbandry for the former, and agriculture, food and beverage for the latter.

## Results and Discussion

In a previous publication, we reported on the development of a highly selective and sensitive self-powered glucose sensor based on a capacitive biofuel cell circuit^34–36^. We demonstrated, for the first time, a novel, free-standing biosensor that is capable of sensing glucose and generating electrical power simultaneously for powering a digital device, such as a glucometer. In this report, we show that the disparate electronic components that constitute the sensor - the biobattery, the capacitive circuit and the indicator, can be completely integrated to form a fully-flexible glucose-indicating decal that can be adhered onto a surface of interest. This is accomplished by integrating the biofuel cell glucose sensor circuitry into our nanocellulose printed circuit board technology (PCB).

Nanocellulose sheets are highly flexible, mechanically stable and strong, and hydrophilic. They range from transparent to translucent, are permeable to gases and liquids, while being chemically-inert, biocompatible and biodegradable^37–39^. The nanocellulose sheets we process in-house are considerably thin (10 µm and below), flexible and resilient to many types of processing conditions. Nanocellulose PCB are formed via the solution-processed plating of metals on microns-thin nanocellulose to create electrodes and wires that form the basis of a PCB. Furthermore, we have developed a low temperature soldering process that allows us to easily solder standard surface mount components onto the nanocellulose PCB.

**Figure 1:**
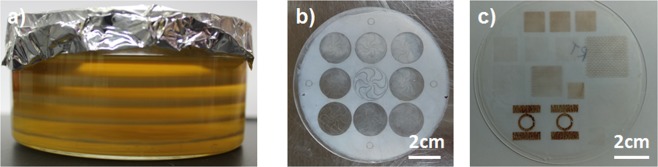
(**a**) Nanocellulose pellicles grown over a 5 cycle period. (**b**) A nanocellulose pellicle on a glass wafer dried into a sheet. (**c**) Inkjet-printed images on nanocellulose.

To construct our flexible, self-powered, self-adhering glucose sensor, we begin with the growth and formation of our nanocellulose sheets. Nanocellulose pellicles are grown via a fermentation process at 30 °C where cellulose chains are produced as *Gluconacetobacter xylinus* consumes simple sugars and polyols. The cellulose chains aggregate to form fibers which in turn aggregate to form millimeter-thick cellulose gel pellicles at the air-water interface, as shown in Fig. 1a. These pellicles, when dried, form microns-thick sheets (Fig. 1b), which are amenable to solution-processing, such as inkjet printing as shown in Fig. 1c. AFM performed on the sheets, depicted in Fig. S1a in the Supporting Information, reveals a porous network of nanocellulose fibers of around 50 nm in diameter, some of them bundled to form thicker cables. Profilometry measurements performed on the sheets show that the sheets range from 0.5 µm to 20 µm. The sheets we used in this report average around 3 µm, as measured in Fig. S1b in the Supporting Information. The porosity of the cellulose sheet is critical, as it allows the infusion of the palladium catalyst ink that is used as a precursor to the electroless plating of metals onto the nanocellulose sheet. There are very few reports of the direct use of microbially-grown sheets of nanocellulose for the fabrication of flexible electronics. To date, many engineering applications of bacterial nanocellulose rely on the maceration of the BNC pellicle, and the subsequent incorporation of the homogenized nanofibrils into a casted film or composite. Nonetheless, there are immense benefits in maintaining the original pellicle structure, while being able to tune its properties *in situ*.

Another advantage is the self-adhering nature of micron-thin sheets of microbial nanocellulose. Dry nanocellulose can be laminated onto many surfaces simply by moistening the sheet, laying it on the surface on interest and allowing it to dry. A strong adhesive bond can be formed between nanocellulose sheets and the surfaces of many materials, such as polymers and inorganic materials, and can only be removed either by moistening or scratching. The strong bond between surfaces is believed to originate from hydrogen bonds from the –OH terminated groups that decorate the cellulose polymer chains^40^. The flexible nature of the sheets allows our nanocellulose electronics to conform onto any surface and remain attached to the location, with intention that the completed nanocellulose biofuel cell-sensor can be directly attached to the skin without the need for any adhesive. Indeed, we have found nanocellulose sheets remaining attached on the skin for over 12 hrs if not inordinately disturbed.

**Figure 2:**
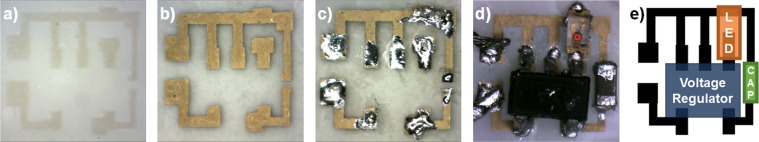
(**a**) Ink-jet print pattern on nanocellulose with a palladium-based catalyst ink. (**b**) Electrolessly-plated a metallic wiring diagram on nanocellulose based on the ink pattern in (**a**). (**c**) Field’s Metal solder coated on the metallic wiring structure. (**d**) Surface mount components welded on the nanocellulose sheet with the solder, with the device wired up and in operation. (**e**) Circuit layout of the resulting electronic device.

To create the nanocellulose PCB, we first used an ink-jet printer to print patterns consisting of palladium-based aqueous ink that serves as catalyst. An ink print on nanocellulose is shown in Fig. 2a. The ink print nanocellulose is plated subsequently with copper, nickel, and gold via electroless plating technique to result in the metallic wiring diagram as shown in Fig. 2b. Our electroless plating process differs from the processes used for fabricating standard flexible PCBs, in which a copper sheet is first laminated onto a plastic sheet, and the copper is subsequently etched to the desired pattern. We have also developed a low temperature soldering process which uses a low melting-point, low toxicity metal alloy - Field’s Metal, to allow surface mount components to be welded onto our nanocellulose PCB. The solder is first coated on the electroless-plated wiring structure on the nanocellulose (Fig. 2c), then the nanocellulose sheet is placed on a hot plate to re-melt the solder, and electronic surface mount components are laid on the solder. The device is then cooled, allowing the components to be welded on the nanocellulose sheet, resulting in the nanocellulose PCB as shown in Fig. 2d.

The charge pump circuit was used to enable the biofuel cell to power a small electronic device while simultaneously monitoring glucose concentration levels. As shown in Fig. 2e, the charge pump circuit consists a voltage upconverter (Seiko S-882Z18), a red LED and a 2.7 μF capacitor. The voltage upconverter is required as the biofuel cell typically has an output voltage of between 0.3 V to 0.5 V and therefore needs to be upconverted to at least 1.5 V in order to power the LED. The biofuel cell consists of MWCNTs sheets (Buckypaper, with an AFM micrograph in Fig. S1c in Supporting Information) functionalized with 1-pyrenebutanoic succinimidyl ester, a heterobifunctional crosslinker which results in π–π stacking of the pyrene moieties on MWCNTs sidewalls and subsequently forms a peptide bond with the lysine group of the enzyme, thereby crosslinking the MWCNTs and the biocatalyst of interest. The anode comprised covalently attached pyrroloquinoline quinone glucose dehydrogenase (PQQ-GDH) onto the 1-pyrenebutanoic succinimidyl ester (PBSE) modified MWCNTs for glucose oxidation. For the cathode, bilirubin oxidase (BOD) was used as the oxygen selective enzyme. Figure S2 in the Supporting Information shows the cyclic voltammagrams of the bioanode and the biocathode in the presence and absence of glucose and oxygen, respectively. In the presence of glucose, a bio-catalyzed oxidation occurs at the anode at an onset potential of −0.22 V accompanied by the release of electrons, which are captured at the cathode where oxygen is reduced to water at an onset potential of 0.59 V, thereby producing electrical current.

**Figure 3:**
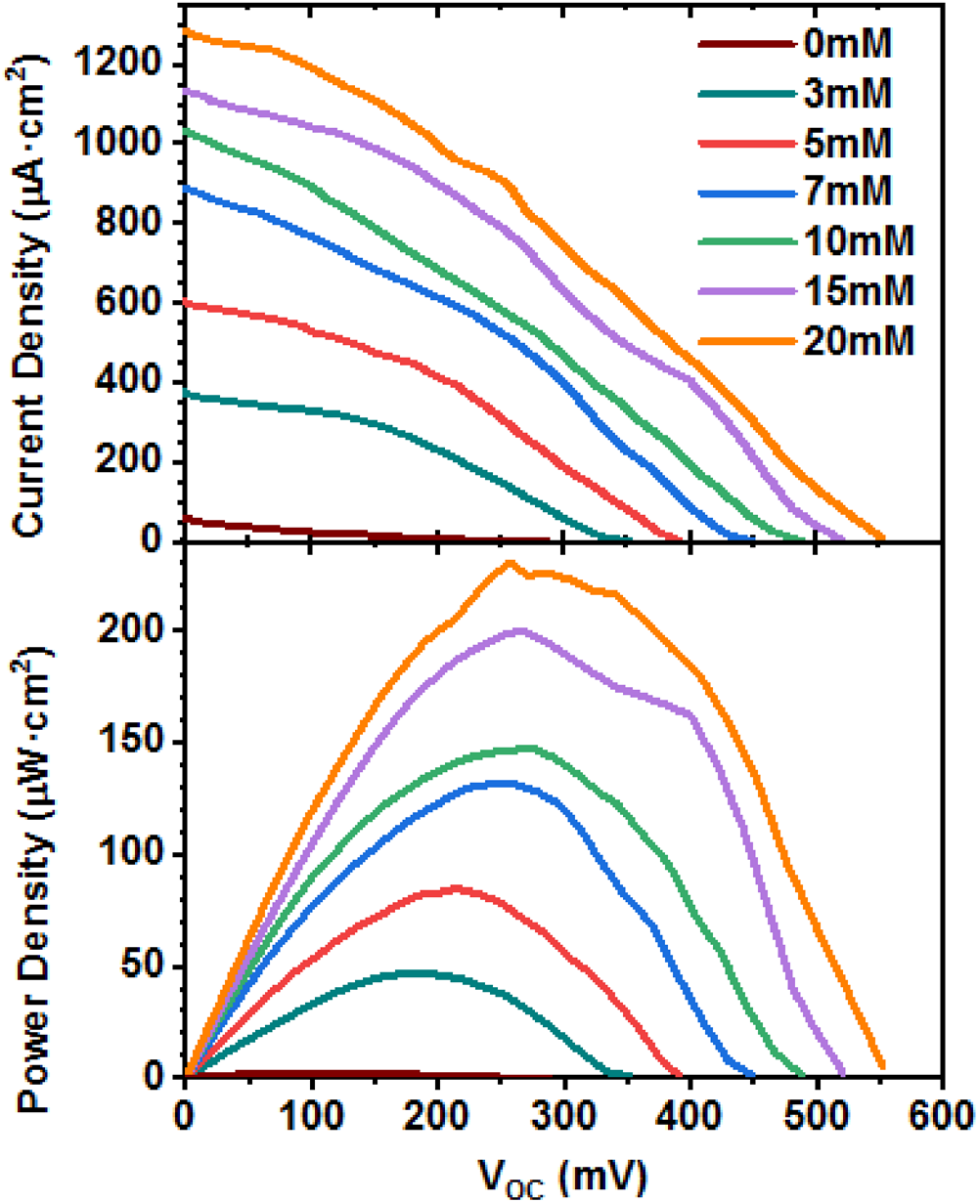
(**a**) Current-voltage polarization curves (top) and power versus current density (bottom) curves of the glucose biofuel cell at different external loads in increasing glucose concentration.

The performance of the biofuel cell operating in various glucose concentrations is depicted in Fig. 3. The polarization curve in the top of Fig. 3 shows that with increasing glucose concentration, the voltage and current density increased from the open circuit voltage of 0.35 V–0.55 V; and short current density of 0.38 mA–1.29 mA/cm^2^. These results are consistent with previous results reporting self-power glucose biosensors made with MWCNTs-laccase as the biocathode^41–43^. The maximum power density of 230.29 µW/cm^2^, at a cell voltage of 0.26 V is obtained when operating in 20 mM glucose (bottom of Fig. 3). At very low glucose concentration (3 mM), the open circuit voltage, short circuit current and power densities were observed to be 0.35 V, 0.38 mA/cm^2^ and 47.29 μW/cm^2^ respectively, at a cell voltage of 0.18 V.

The target application for the biofuel cell glucose indicator is for surface mount deployment on an object, such as the human body where fuel, the analyte of interest, can be wicked through both the nanocellulose PCB and biocatalysts immobilized Buckypaper without the use of any external pumps and electrical power. This arrangement enables the oxidation of the glucose molecules in the fuel sample at the bioanode as well as the immediate and direct electron transfer from PQQ-GDH to the current collector. The released electrons travel through the external circuitry and recombine at the biocathode, where BOD reduces oxygen to water. The porosity of the nanocellulose PCB and Buckypaper allowed a higher volume of analyte sample to be wicked and retained in the bioelectrodes for a long duration to enable stable power production in order to power small electronic devices connected to the body. In this context, the charge pump circuit was designed to be powered by the biofuel cell. Moreover, the single, unstacked biofuel cell deliver voltages between 0.3 V to 0.5 V (glucose concentration dependent). The charge pump circuit used required an input voltage greater than 0.27 V to operate, which the single biofuel cell was capable of providing, when placed in a glucose and oxygen containing buffer or a cotton mat saturated with glucose. The integration of the constructed charge pump circuit amplified the nominal voltage delivered by the biofuel cell to 1.8 V by gradually charging the startup capacitor and raising its voltage.

**Figure 4:**
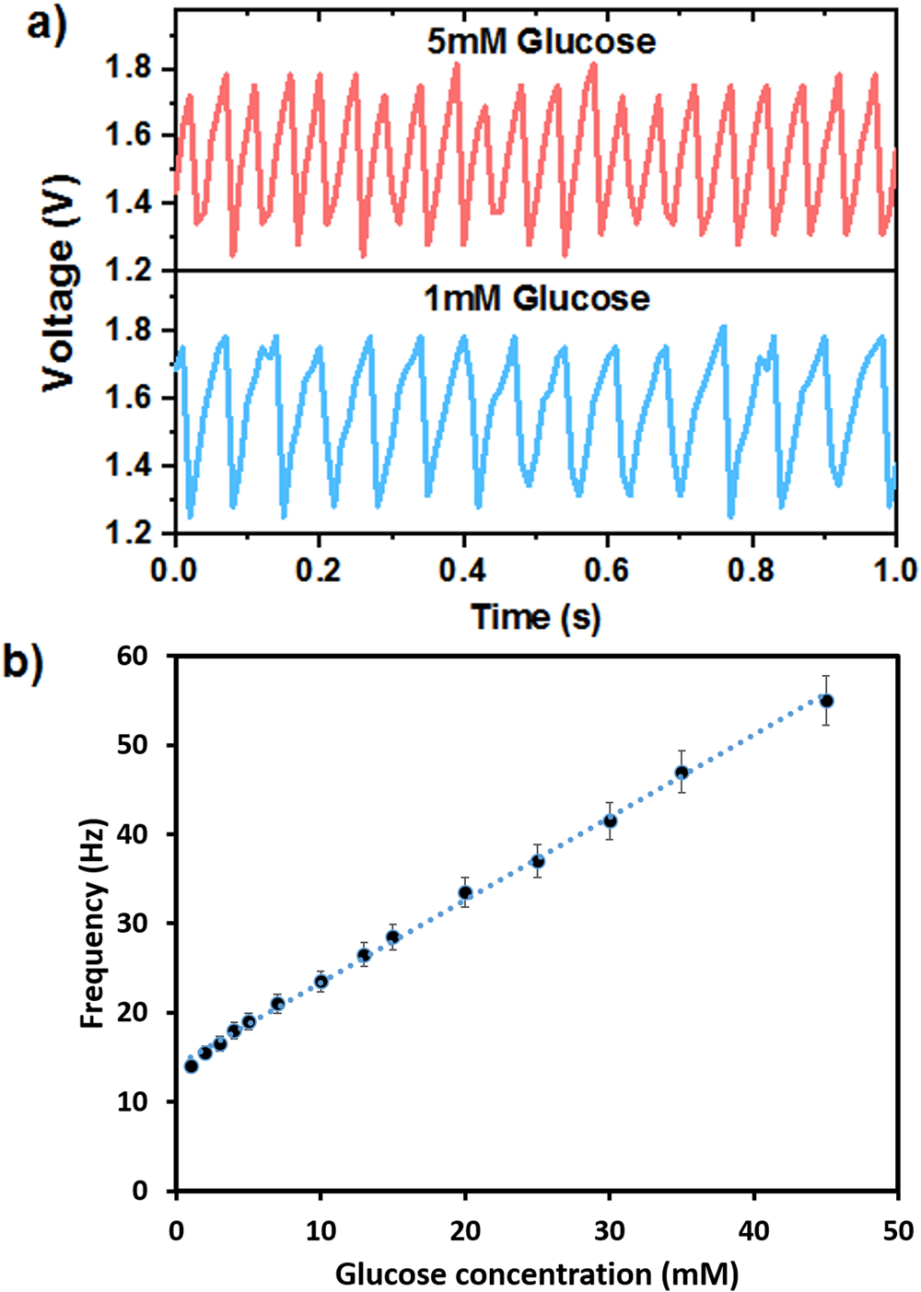
A Steady-state frequency – time responses obtained by glucose biofuel cell to 1 mM and 5 mM glucose in 0.1 M PBS solution at the 2.7 μF capacitor. (**c**) Calibration curve: frequency versus glucose concentration. Error bars represent standard errors of the mean triplicate values.

Figure 4a depicts the performance characteristics of the 2.7 μF capacitor integrated with glucose biofuel cell. The redox reaction at the bioelectrodes resulted in the generation of electrical power, which is subsequently fed into a charge pump circuit to charge the capacitor. Once the capacitor is fully charged, the charge pump circuit discharges the capacitor to a voltage of ca. 1.2 V–1.4 V. Furthermore, there was a direct correlation observed between the charge and discharge cycle of the capacitor and glucose concentration as shown in Fig. 4b, thereby confirming the oxidation of glucose at the bioanode. These results demonstrated that the biofuel cell glucose indicator exhibited excellent linear relationship between the charge/discharge and glucose concentration. A dynamic linear range of 1 to 45 mM glucose was observed with a correlation coefficient of 0.998 with a sensitivity of 11.56 Hz/mM cm^2^.

**Figure 5:**
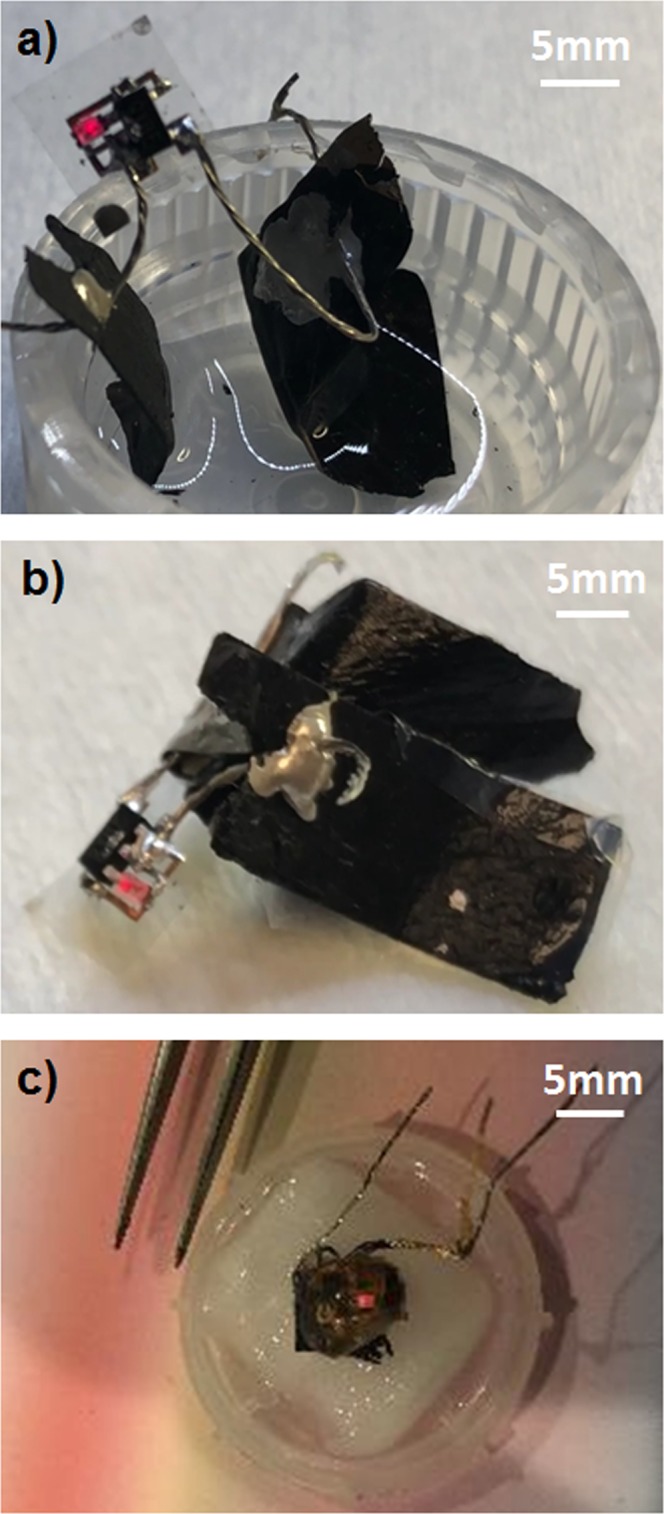
(**a**) A flexible self-powered glucose indicator immersed in a glucose solution; device is in operation with the LED flashing red. Video is available in the Supporting Information. (**b**) A flexible self-powered glucose indicator with the biofuel cell electrodes sandwiching a nanocellulose sheet soaked with glucose solution, showing the autonomous operation of the device in an isolated environment. Video is available in the Supporting Information. (**c**) Final form of the flexible self-powered glucose indicator reduced to a 5 mm × 5 mm square in operation.

Having described the individual units that comprise the full device, our final step is to link the flexible PCB and soft biofuel cell up to complete the construction of the fully flexible biofuel cell glucose indicator. Initially, we found it difficult to attach solder to the CNT electrodes of the biofuel cell but did manage to find an alternative solution for integrating the MWCNTs bioelectrodes. First, we soldered the wires to the nanocellulose PCB and then the wires were simply threaded into the Buckypaper bioelectrodes. The electrical contacts between the wires and the bioelectrodes were strengthened via the coating of silver paint where the wires cross the electrodes. Figure 5a, depicts the charge pump circuit integrated with the biofuel cell operating on 3 mM glucose, wherein the LED is powered on by the biofuel cell as it oxidizes and reduces glucose and oxygen, respectively. An accompanying video showing the device in real-time operation is included in the Supporting Information. When the device was immersed in water, the LED did not light up, as shown in Fig. S3 in the Supporting Information. Figure 5b proves both energy and operational autonomy of our device with a standalone device operating is isolation. Based on the experiments, we conclude that the device operated successfully as a visual indicator of the presence of glucose without the need for additional apparatus. We can imagine the device operating autonomously in a specific location, and that a user can easily detect the presence of the indicator visually through the blinking LED, and hence confirm the presence of glucose, without the need of additional tools. Moreover, additional work has been done to shrink the device to a tiny 6 mm × 6 mm square, as depicted in Fig. 5c, allowing the device to be extremely compact, and thereby user and location friendly. Using our device, and by modifying the type of enzyme on the electrode, we believe that other analytes can possibly be interrogated, such as lactate, and a variety or sugars and polyols.

## Conclusion

We have developed a flexible, nanocellulose-based, highly-sensitive, glucose indicator that is capable of detecting glucose while powering an indicator (LED) simultaneously. This device was built on our specially developed nanocellulose printed circuit boards and is fully flexible and biocompatible, resulting in an autonomous glucose indicator with the entire structure collapsed into a single half-centimeter-square, flexible decal weighing less than 40 mg. Applications with other analytes for light-weight, imperceptible human-health monitoring applications are envisioned for this technology.

## Methods

### Materials and components

Buckypaper (aggregated multi-walled carbon nanotubes [MWCNTs]) was purchased from Nanotech labs (Yadkinville, NC). 1-Pyrenebutanoic succinimidyl ester (PBSE) (bi-functional crosslinker) was purchased from AnaSpec. Inc. PQQ-GDH was purchased from Toyobo. Co. Ltd. All other chemicals and components, such as bilirubin oxidase (Myrothecium), dimethyl sulfoxide (DMSO), D- (+)-Glucose, potassium phosphate, calcium chloride, and chitosan were purchased from Sigma Aldrich. The S882z charge pump circuit was purchased from Seiko electronics.

### Nanocellulose growth and sheet formation

*Gluconacetobacter xylinus* were cultured in HS medium 1 pH 6.0 at 30 °C. The mother cultures were placed in 50 mL conical tubes for a duration of 10–14 days to enable the cells to proliferate and grow. The inoculum was generated by dislodging the pellicle and dispersing cells via repeated vortexing at maximum speed for 30 s to separate the precipitated pellicle and/or diffuse cellulose from the suspended bacteria cells. Aliquots of 1 mL of the bacteria suspension were added to sterile 100 mm crystallization dishes containing 50 mL of HS medium and were covered with sterile aluminum foil and maintained in an incubator. Fresh medium was added every week for a total of six weeks to the surface of the basal pellicle to allow for the formation of six uniform pellicles.

To decontaminate the nanocellulose pellicles, the pellicles were incubated in 0.5 M NaOH solution at 90 °C in for 1 hour followed by extensive rinse with 18 mΩ water to remove NaOH and proteins/bacteria. The harvested pellicles were stored in water comprising 0.02% sodium azide to prevent the growth of microbials. The nanocellulose pellicles were placed on 10 mm glass wafers and allowed to dry at room temperature.

### AFM characterization of nanocellulose sheets and bucky paper

Atomic Force Microscopy was performed on both the nanocellulose sheets and the buckypaper to characterize their morphology. A Digital Instruments Dimension 3100 AFM was used to characterize the nanocellulose samples, while a JPK Instruments NanoWizard® 4a AFM was used for the Buckypaper. For the Digital Instruments AFM, we used triangular Si_3_N_4_ microcantilevers (Nanoprobe, Veeco) and for the JPK Instruments AFM, we used Tap 300A-1-G probe tips from Budget Sensors.

### Thickness measurements of nanocellulose sheets

Thickness measurements of the nanocellulose sheets were performed on a KLA-Tencor Alpha-Step D-500, on which selected areas on each wafer were scratched with a tweezer to reveal the underlying wafer from which the profilometry measurement was performed.

### Electroless plating on nanocellulose sheets

Inkjet printing of Pd catalyst ink patterns was performed with a FujiFilm Dimatix DMP-2831 Materials Printer. Cataposit 44 (Rohm & Haas), used as received, was diluted 1:6 with 11% hydrochloric acid and filtered into a DMC-11610 cartridge using a 0.2 µm Nalgene PTFE syringe filter. The platen and cartridge temperatures were maintained at 37 °C and room temperature, respectiviley. The printing resolution was set at a resolution of 1270 DPI and the jetting voltage was maintained between 15–35 V using 4 jets. After printing, the nanocellulose sheet was immersed in DI water to remove the acid in the ink, and the printed nanocellulose sheet was peeled off the glass wafer it was mounted on. The peeled sheet was remounted while still in DI water onto a transparency sheet, then removed from the DI bath and air-dried. Upon drying, double-sided tape was attached to the edges of the transparency to secure the nanocellulose sheet.

Three layer of different metals, copper, nickel and gold were plated onto the catalyst patterns by immersing the mounted transparency sheet into specific chemical baths. Plating of copper was carried out using Cuposit 328 electroless copper plating solution at 55–60 °C; plating of nickel was carried out using Duraposit SMT88 electroless nickel plating solution at 88 °C; and gold, with Aurolectroless 520 gold plating solution at 88 °C. Upon the completion of each plating step, the sample was soaked in water (three times) to remove the residual electroless bath. After all the plating steps, the samples were left overnight to air-dry.

### Soldering of surface mount electronics and electrodes on nanocellulose

Field’s Metal (Roto144F, Rotometals), a low melting point, low toxicity metal alloy consisting of bismuth, tin and indium, was used as the solder. To connect the surface mount devices to the plated electrodes on the nanocellulose sheet, a solder flux was first applied to the metal surface to ensure wetting of the solder on the gold. Field’s Metal was then applied to the treated metal surface. After applying Field’s Metal to selected areas of the plated metal layer, the entire sheet was placed on a hot plate at 80 °C, allowing the solder to re-melt. The surface mount electronics were then placed on the melting solder pads. After placement, the sheet is taken off the hot plate and cooled, whereupon the solder cools and the electronics were welded firmly onto the sheet. The assembled nanocellulose PCB was then tested for electrical continuity.

### Enzyme activation of the buckypaper

Each of the buckypaper electrodes were rinsed with isoproponal (IPA) and were subsequently placed in PBSE (1 mM) solution crosslink the MWCNTs to the enzyme via ‘π–π’ stacking and peptide bond. The bioelectrodes were prepared by dissolving PQQ-GDH (1 mg/ml) in 10 mM PBS (pH 7.0) containing 1 mM CaCl_2_ (pH 7.0) and BOD in 10 mM PBS. After the immobilization of glucose and oxygen selective enzymes on the respectivie bioelectrodes, the bioelectrodes were coated with chitosan to enhance the stability of the immobilized enzymes. These bioelectrodes were used in the development of the biofuel cell.

### Biofuel cell electrical testing

The glucose biofuel cell was built using the as-fabricated MWCNTs modified with PQQ-GDH as the bioanode, and MWCNTs modified with BOD as the biocathode. All experiments were performed in 5.0 mM glucose (pH = 7.4)) at 22 °C. The PQQ-GDH catalyzes the oxidation of glucose, whereas BOD catalyzes oxygen reduction. The polaraization outputs of the biofuel cell were obtained using various loads connected directly in parallel with the cell while operating on various glucose concentrations. The voltage and current readings were recorder with a Fluke 87 V True RMS multimeter. An Agilent DSO6014A Oscilloscope was used to capture the charge/discharge characteristics of the 2.7 μF capacitor.

## Supplementary information

Supplementary Figures
