# Supplementary material for: A Fully-Flexible Solution-Processed Autonomous Glucose Indicator
Source: Sci Rep. 2019 May 6;9:6931. doi: 10.1038/s41598-019-43425-x (PMC6502809; doi:10.1038/s41598-019-43425-x)
Supplement: Supplementary file 1 — Supplementary Figures [file 41598_2019_43425_MOESM1_ESM.docx]

A Fully-Flexible Solution-Processed Autonomous Glucose Indicator

Jonathan D. Yuen,^1^ Ankit Baingane,^2,3^ Md Qumrul Hasan,^2,3^ Lisa C. Shriver-Lake,^1^ Scott A. Walper,^1^ Daniel Zabetakis,^1^ Joyce C. Breger,^1^ David A. Stenger,^1^ and Gymama Slaughter^2,3^*

1. Center for Bio-Molecular Science and Engineering, U.S. Naval Research Laboratory, Washington, DC 20375, USA
2. Frank Reidy Research Center for Bioelectrics and Department of Electrical & Computer Engineering, Old Dominion University, Norfolk, VA 23529, USA
3. Department of Computer Science and Electrical Engineering, University of Maryland Baltimore County, Baltimore, MD 21250, USA

* gslaught@odu.edu

# Figure S1


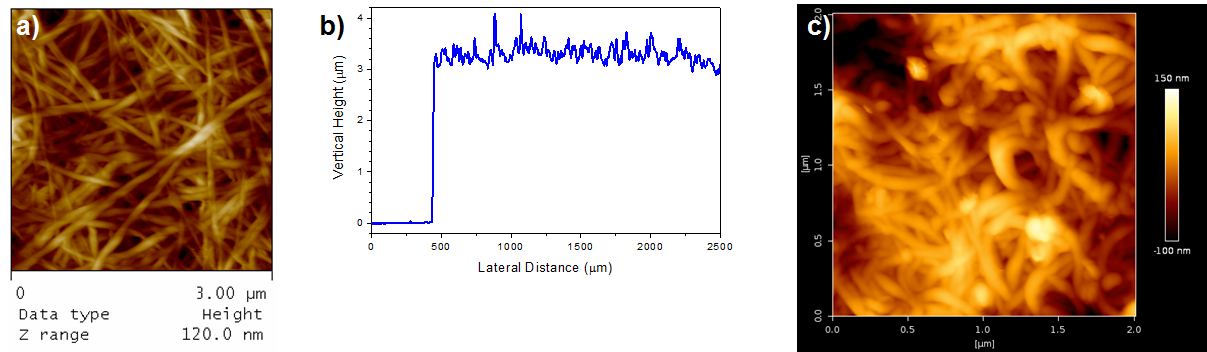


Figure S1. a) AFM micrograph showing the fibrous morphology of the nanocellulose sheet. b) Profilometer profile of a typical nanocellulose sheet. c) AFM micrograph depicting a Buckypaper electrode post enzyme passivation.

# Figure S2


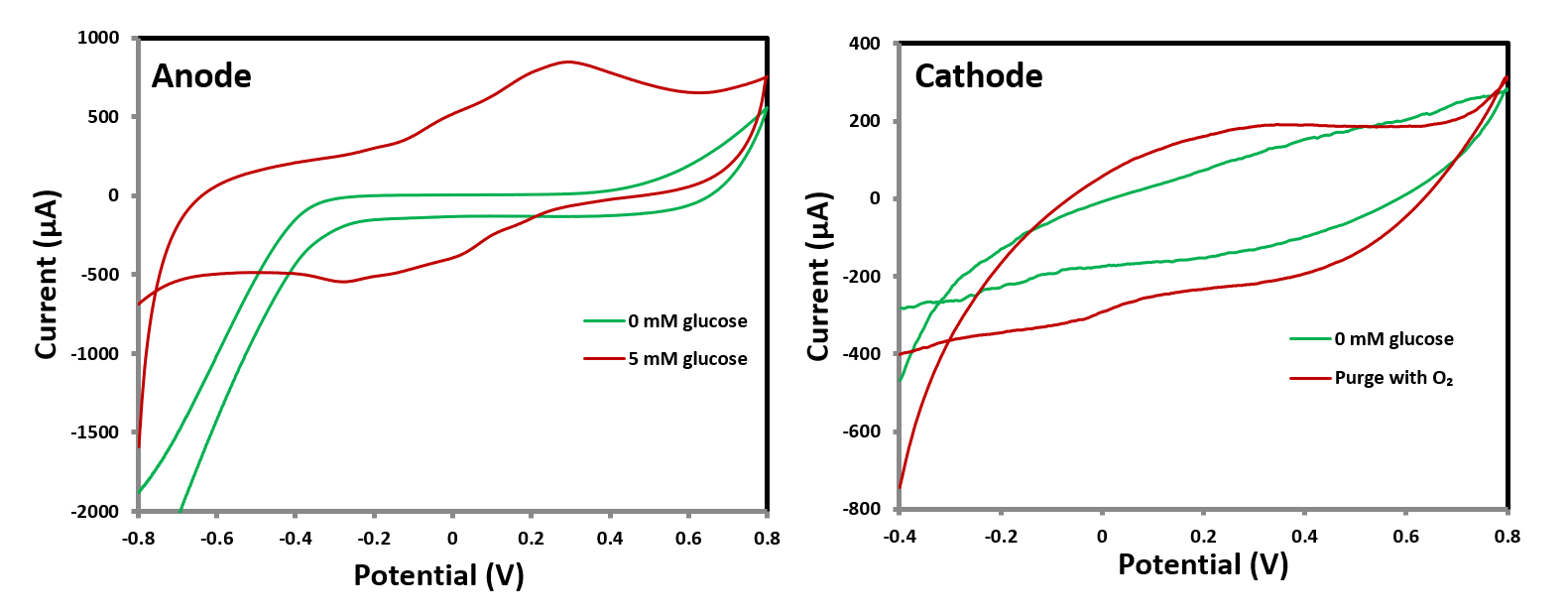


Figure S2. Cyclic voltammograms of bioanode (left) both in PBS (0 mM glucose) as a control (blank), and with the addition of 5 mM of glucose, and the biocathode (right) in PBS and with the PBS purged with oxygen. In both cases, the addition of the analyte results in an increase in the oxidation and the reduction current.

# Figure S3


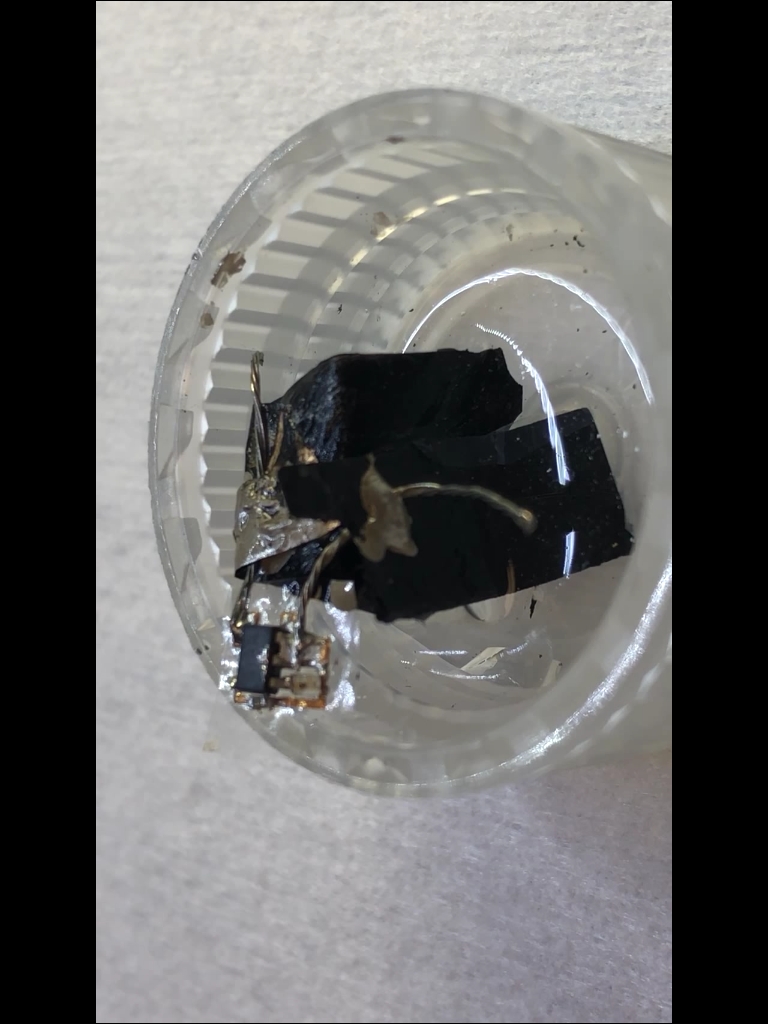


Figure S3. A flexible self-powered glucose indicator immersed in a water; device cannot operate and the LED does not flash red.
